# Purinergic P2Y12 Receptor Activation in Eosinophils and the Schistosomal Host Response

**DOI:** 10.1371/journal.pone.0139805

**Published:** 2015-10-08

**Authors:** Valdirene S. Muniz, Renata Baptista-dos-Reis, Claudia F. Benjamim, Hilton A. Mata-Santos, Alexandre S. Pyrrho, Marcelo A. Strauch, Paulo A. Melo, Amanda R. R. Vicentino, Juliana Silva-Paiva, Christianne Bandeira-Melo, Peter F. Weller, Rodrigo T. Figueiredo, Josiane S. Neves

**Affiliations:** 1 Institute of Biomedical Sciences, Federal University of Rio de Janeiro, Rio de Janeiro, RJ, Brazil; 2 Pharmacy School, Federal University of Rio de Janeiro, Rio de Janeiro, RJ, Brazil; 3 Institute of Medical Biochemistry, Federal University of Rio de Janeiro, Rio de Janeiro, RJ, Brazil; 4 Institute of Biophysics Carlos Chagas Filho, Federal University of Rio de Janeiro, Rio de Janeiro, RJ, Brazil; 5 Division of Infectious Diseases and Allergy and Inflammation, Harvard Medical School, Beth Israel Deaconess Medical Center, Boston, MA, United States of America; 6 Institute of Biomedical Sciences/Unit of Xerem, Federal University of Rio de Janeiro, Rio de Janeiro, RJ, Brazil; Fundação Oswaldo Cruz, BRAZIL

## Abstract

Identifying new target molecules through which eosinophils secrete their stored proteins may reveal new therapeutic approaches for the control of eosinophilic disorders such as host immune responses to parasites. We have recently reported the expression of the purinergic P2Y12 receptor (P2Y12R) in human eosinophils; however, its functional role in this cell type and its involvement in eosinophilic inflammation remain unknown. Here, we investigated functional roles of P2Y12R in isolated human eosinophils and in a murine model of eosinophilic inflammation induced by *Schistosoma mansoni* (*S*. *mansoni*) infection. We found that adenosine 5’-diphosphate (ADP) induced human eosinophils to secrete eosinophil peroxidase (EPO) in a P2Y12R dependent manner. However, ADP did not interfere with human eosinophil apoptosis or chemotaxis *in vitro*. *In vivo*, C57Bl/6 mice were infected with cercariae of the Belo Horizonte strain of *S*. *mansoni*. Analyses performed 55 days post infection revealed that P2Y12R blockade reduced the granulomatous hepatic area and the eosinophilic infiltrate, collagen deposition and IL-13/IL-4 production in the liver without affecting the parasite oviposition. As found for humans, murine eosinophils also express the P2Y12R. P2Y12R inhibition increased blood eosinophilia, whereas it decreased the bone marrow eosinophil count. Our results suggest that P2Y12R has an important role in eosinophil EPO secretion and in establishing the inflammatory response in the course of a S. mansoni infection.

## Introduction

Parasitic helminth infections, which are prominent agents of disease in resource-limited regions, are the most common cause of persistent eosinophilia, a prominent feature of immune responses to *Schistosoma mansoni* (*S*. *mansoni)* [1]. Eosinophils are leukocytes that contain an abundance of large cytoplasmic granules that are ultrastructurally unique due to their internal crystalline cores. These granules store a variety of preformed proteins, including four cationic proteins, eosinophil cationic protein (ECP), major basic protein (MBP), eosinophil peroxidase (EPO), and eosinophil-derived neurotoxin protein (EDN); hydrolytic enzymes; and more than three dozen cytokines (including IL-13, IL-4, IL-5, IL-10, IL-6, TNF-α and IFN-γ), chemokines and growth factors that can be rapidly mobilized and released extracellularly [1, 2]. More than two billion people are infected with parasitic helminths worldwide [3]. Although infections by these pathogens are generally not fatal, they are associated with high rates of morbidity [3]. Infections with *S*. *mansoni* cause dramatic increases in eosinophilopoiesis, in the levels of eosinophils in peripheral blood and in the granulomas induced in the host tissues by parasite eggs [4]. The morbidity due to *S*. *mansoni* infections arises from the granulomatous, immune-mediated response to eggs that become trapped in the host liver and gut after being released by fecund females residing in the mesenteric venous plexus [4]. Eosinophils have long been traditionally thought to function solely as terminal effector cells in Th2 immune responses, including exerting helminthotoxic activity against parasites. Eosinophils can kill schistosomula *in vitro* in the presence of antibody and/or complement [5]. However, the function(s) of eosinophils in parasitic infections *in vivo* remains poorly understood. *In vivo*, eosinophils have the capacity to invade dying schistosomes [6], suggesting that they might be an important defense mechanism against the parasite. Eosinophils are also responsible for a considerable amount of the inflammatory pathology accompanying helminth infections. Similarities between the sequelae of hypereosinophilic syndromes, which are syndromes characterized by extremely high levels of eosinophilia [7], and the pathologic consequences of helminth infection suggest a potential role for eosinophils in the immunopathogenesis of these infections. Eosinophils can damage both parasites and their surrounding tissues through a number of different mechanisms, including reactive oxygen species (ROS) generation, release of eosinophil cationic proteins and lipid mediators, such as leukotrienes [8]. However, two different mouse lineages genetically deficient in eosinophils exhibited no prominent alterations in worm burdens or granuloma formation during experimental *S*. *mansoni* infections [9], suggesting that eosinophils do not simply function as helminthotoxic killer cells or pro-inflammatory cells. Data collected over the years are beginning to reveal more complex roles for eosinophils, including a more dynamic picture of Th2 immunity, where eosinophils are present very early in the response to Th2-inducing agents and function as antigen-presenting cells contributing to the initiation of Th2 immunity [10]. Recently, we identified the secretion competence of cytolytically released cell-free eosinophil granules (as found in the lesions around helminths) that, once deposited extracellularly in tissues, might themselves enhance Th2 immune responses in the absence of intact eosinophils [11–13].

Purinergic P2 receptors are expressed in many tissues and organs and are activated by extracellular nucleotides, such as adenosine triphosphate (ATP), adenosine diphosphate (ADP), uridine 5’-diphosphate (UDP) and uridine 5’-triphosphate (UTP), which are released as a consequence of cell damage, cell stress, bacterial infection or other noxious stimuli. After tissue injury, intracellular nucleotides can be released into the extracellular environment, acting as proinflammatory endogenous signaling molecules (danger-associated molecular pattern–DAMPs—or *alarmins)* capable of functioning as both chemoattractants and activation signals for immune cells [14, 15]. According to their molecular structure, P2 receptors are divided into 2 subfamilies–P2X and P2Y receptors. The P2X receptors are ligand-gated channels, whereas P2Y receptors are G- protein-coupled, seven-membrane-spanning receptors. Several studies indicate that nucleotides play an important role in immune response modulation through their action on multiple cell types, including monocytes, mast cells, dendritic cells, neutrophils and eosinophils [15–17]. Human eosinophils express several mRNAs that encode P2X and P2Y purinergic receptor subtypes, P2Y1, P2Y2, P2Y4, P2Y6, P2Y11, P2Y14, P2X1, P2X4 and P2X7; however, the activation of these purinergic receptors and their capacities to mediate different eosinophil responses are not fully known [18]. We have recently reported the expression of the P2Y12 receptor (P2Y12R) on human eosinophils and cell-free human eosinophil granules [12]. However, the functional roles of P2Y12R and its contributions to eosinophilic inflammation and host defense remain to be elucidated. P2Y12R is a Gi/o coupled receptor that is classically responsive to ADP. The key roles for ADP and its receptors in vascular biology and platelet aggregation *in vivo* are well established by studies with P2Y1 and P2Y12 receptor-null mice and are validated in humans by the clinical efficacy of P2Y12 antagonists (e.g., clopidogrel) as antiplatelet agents [19]. Recently, P2Y12R was identified as a receptor sensitive to leukotriene E4 (LTE4) playing a prominent role in the mediation of LTE4–induced pulmonary inflammation [20]. Thus, modulation of P2Y12R might represent a new strategy to control eosinophil activation and degranulation in eosinophilic pathological conditions. The goal of this study is to investigate whether P2Y12R has functional roles in eosinophil activation and in the inflammatory host immune response induced by *S*. *mansoni* infection.

## Materials and Methods

### Ethics statement

All protocols and experimental procedures involving animals were approved by the Committee for Ethics in Animal Experimentation (CEUA) at Federal University of Rio de Janeiro (license number DFBCICB 043). Mice were sacrificed by anesthesia (pentobarbital, 40 mg/kg and xylazine, 2.5 mg/kg, intramuscularly) followed by cervical dislocation. All efforts were made to minimize suffering during the procedures in this project. All animal experiments were conducted in accordance with Brazilian Federal Law number 11.794, which regulates the scientific use of animals, and IACUC guidelines. Human peripheral blood for eosinophil purification was obtained with written informed consent from healthy donors under protocols approved by the Clementino Fraga Filho Hospital Ethical Committee (Federal University of Rio de Janeiro) (license number 190/09).

### Eosinophil purification and stimulation

Eosinophils were purified from the blood of healthy donors as previously described [11]. The viability and purity of freshly isolated eosinophils were >99% as analyzed by trypan blue exclusion and Panoptic kit staining, respectively. (250,000/250 μL) were resuspended in RPMI 1640 plus 0.1% ovalbumin (without phenol red) (Sigma, St. Louis, MO, USA) and stimulated with ADP (0.1–100 nM) (Sigma, St. Louis, MO, USA). Treatments with MRS2395 (10 μM) (Sigma, St. Louis, MO, USA), a selective P2Y12R antagonist, or with MRS2179 (10 μM) (Sigma, St. Louis, MO, USA), a selective P2Y1R antagonist, were performed for 15 min prior to stimulation with ADP (100 nM) for 1 h at 37°C. Thereafter, supernatants were collected and stored at −20°C for further EPO quantification. Drugs were diluted in DMSO at a final concentration of <0.01%, which had no effect on eosinophil secretion.

### Assay of eosinophil-secreted EPO

EPO activity was measured in the supernatants using a colorimetric assay [21]. Briefly, 20 μL of the supernatants were incubated with 100 μL substrate solution (50 mM Tris-HCl, 1 mM H_2_O_2_, 2 mM O-phenylenediamine dihydrochloride (OPD) (Sigma, St. Louis, MO, USA) and 0.1% Triton X-100, pH = 8). The reaction was stopped after 30 minutes of incubation by the addition of 100 μL 4 M H_2_SO_4_. The plate was read at 492 nm.

### Bone marrow derived eosinophils

With slight modifications, eosinophils were differentiated *in vitro* from mouse bone marrow cells, as previously described [22]. Briefly, bone marrow cells were collected from femurs and tibiae of wild-type BALB/c mice with RPMI 1640 (Sigma-Aldrich, St. Louis, MO, USA) without FBS. After red blood cell lysis, cells were cultured at 10^6^ cells/ml in RPMI 1640 containing 20% FBS (VitroCell, Campinas, SP, Brazil), 100 IU/ml penicillin, 10 μg/ml streptomycin, 2 mM glutamine (Sigma-Aldrich, St. Louis, MO, USA), 1 mM sodium pyruvate (Sigma-Aldrich, St. Louis, MO, USA), 1% non essential amino acid solution (MEN) (Sigma-Aldrich, St. Louis, MO, USA), 100 ng/ml stem cell factor (PeproTech, NJ, USA), and 100 ng/ml FLT3 ligand (PeproTech, NJ, USA) from days 0 to 4. On day 4, the stem cell factor and FLT3 ligand were replaced by IL-5 (10 ng/ml; Peprotech, NJ, USA). On day 14, eosinophils were enumerated and labeled for flow studies.

### Flow cytometry of eosinophils

Human or bone marrow derived eosinophils were fixed in 4% paraformaldehyde (PFO) and permeabilized for 5 min on ice with 0.1% saponin (Sigma, St. Louis, MO, USA) before incubation with the primary antibody (Ab), control Ab or primary Ab premixed with the blocking peptide (1 h). Next, the cells were washed and incubated with the respective FITC-conjugated secondary Abs for 15 min on ice in the continued presence of saponin. After staining, the eosinophils were fixed in a buffer containing 4% paraformaldehyde (PFO). Analyses were performed on a FACScan with CELLQUEST software (BD Biosciences, CA, USA). For labeling, rat anti-Siglec-F phycoerythrin(PE)-conjugated antibody (1:100, BD Pharmingen, CA, USA; Siglec-F is a murine eosinophil marker), rabbit anti-P2Y12R polyclonal Ab (1:100, Alomone Labs, Jerusalem, Israel) (directed against the carboxy-terminal intracelullar domain of the receptor) or the anti-P2Y12R Ab premixed with the blocking peptide were used. Anti-rabbit Ab conjugated to fluorescein (FITC) (1:500, Jackson ImmunoResearch, PA, USA) was used as a secondary Ab.

### Fluorescence Microscopy

Eosinophils were stained as described for flow cytometry. Subsequently, cells were incubated with DAPI (1 μg/ml) and coverslipped. In a different set of experiments, murine eosinophil-enriched suspensions were cytocentrifuged and fixed in a buffer containing 4% PFO. Thus, cells were permeabilized for 5 min with 0.1% saponin (Sigma, St. Louis, MO, USA) before the incubation with rabbit anti-P2Y12R polyclonal Ab or the primary Ab premixed with the blocking peptide (1:100, Alomone Labs, Jerusalem, Israel) (directed against the carboxy-terminal intracelullar domain of the receptor) and with the rat anti-Siglec-F phycoerythrin(PE)-conjugated antibody (1:100, BD Pharmingen, CA, USA; Siglec-F is a murine eosinophil marker). Anti-rabbit Ab conjugated to fluorescein (FITC) (1:500, Jackson ImmunoResearch, PA, USA) was used as a secondary Ab. Control or nonimmune Abs were included for all. Fluorescence images were acquired using an Olympus DP72 camera coupled to a BX-53 Olympus microscope (Olympus, Center Valley, PA, USA).

### Detection of apoptotic cells

Classic annexin V labeling was used. Eosinophils were cultured in the presence of recombinant human IL-5 (30 ng/ml) (positive control) or ADP (10–100 nM) or MRS2395 (10 μM) for 24 h, followed by the addition of FITC-labeled annexin V (an early apoptosis marker) (1 μg/mL, Biovision, Mountain View, CA). The cells were incubated for 15 min on ice and immediately analyzed on the flow cytometer (BD Accuri C6, BD Biosciences).

### Eosinophil migration assay

Human eosinophils were resuspended in RPMI 1640 plus 0.1% ovalbumin and added (3x10^5^ cells in 100 μL) in duplicate to the upper chambers of 24-well transwell plates (Corning Glass, Corning, MA, USA) with a 5 μm pore size. ADP (100 and 10 nM) or eotaxin (20 ng/mL) were diluted in 600 μL RPMI 1640 plus 0.1% ovalbumin medium and placed in the lower chambers of the transwell plates. In a different set of experiments, the eosinophils were pre-treated with the compound MRS2395 (a P2Y12R antagonist) or its diluent (DMSO) at 37°C for 15 min or primed for 2 h at 37°C with IL-5 (30 ng/mL) before the transwell system was assembled. The plates were incubated for 3 h at 37°C before recovering, and migrating cells from the lower compartment were counted. The cells were quantitated from duplicate wells by averaging the total events counted in 100 μL using a flow cytometer (BD Accuri C6 –counter mode, BD Biosciences).

### Animals and *S*. *mansoni* infection

C57Bl/6 mice (8 weeks) were percutaneously infected with 60 cercariae of the Belo Horizonte (BH) strain of *S*. *mansoni*. Animals from all groups were sacrificed under anesthesia followed by cervical dislocation after 55 days of infection. Two schistosome independent infection studies were performed with at least 5 animals/each group.

### Parasitological parameters

Hepatic and intestinal tissues were maintained in 4% KOH at room temperature for 12 h, followed by 1 h of incubation at 37°C. The results were expressed as the number of eggs per gram of tissue. Counts were performed in triplicate using light microscopy (100x).

### Histopathology and morphometric analysis

Tissue samples were fixed in 10% buffered formalin and embedded in paraffin. Five-micrometer sections were stained with hematoxylin-eosin or Llewellyn’s Sirus Red (Direct Red 80, CI 35780; Aldrich, Milwaukee, WI) for better eosinophil visualization. Quantitative analysis of the tissue sections and of captured images was carried out using a computer-assisted image analyzer (Scion Image version 4.0.3.2, Scion Corp). All evaluations were performed by two different blinded observers. The area of hepatic granuloma was determined in histological sections from 20 to 30 granulomas per animal, containing central eggs, randomly chosen. Digital photographs were obtained under light microscopy at 400x magnification. To estimate the size of granulomas, we measured their individual areas using the measurement tool of the image analyzer. Eosinophil infiltrates were identified in the granuloma area and expressed as eosinophils/unit area (μm^2^).

### Quantification of cytokines

Cytokine concentrations (IL-4 and IL-13) in the hepatic tissue and plasma were measured using a sandwich enzyme-linked immunosorbent assay technique (ELISA) with capture and detection antibodies according to the instructions of the manufacturer (Peprotech, NJ, USA).

### Hydroxyproline quantification

Hepatic and intestinal tissues were digested as described by Cheever [23]. Livers were maintained in acetone at room temperature until complete dehydration, followed by hydrochloric acid hydrolyzation for overnight incubation at 107°C. Colorimetric assay was then performed using chloramine-T buffer (Sigma, USA), Ehrlich's reagent (Sigma, USA), and perchloric acid (Merck).

### Blood and bone marrow eosinophilia

Blood eosinophilia was analyzed by light microscopy of blood smears after Panoptic kit staining. To evaluate bone marrow eosinophilia, mice were killed and the femurs removed. The bone marrow cavity was flushed with RPMI 1640 (Sigma, St. Louis, MO). The cells were cytocentrifuged and counted under light microscopy after Panoptic kit staining.

### Murine eosinophil-enriched suspensions

Livers from *S*. *mansoni*-infected mice were homogenized, and intact granulomas were allowed to sediment. After multiple washes with RPMI 1640 medium, granulomas were transferred to culture bottles and left overnight 37^°^C in CO_2_ incubator. Eosinophils remain in suspension while other cells (like macrophages) attach to the bottle. After that, medium was collected and centrifuged. Pellets were suspended in medium and cells were counted. The cells were cytocentrifuged and counted under light microscopy after Panoptic kit staining or anti-Siglec-F staining (Siglec-F is a murine eosinophil marker) to determine the percentage of eosinophils in suspension. The nonadherent fraction was composed by 50% eosinophils.

### Tail bleeding test

The tail bleeding test was performed in accordance with Broze and colleagues [24]. After 23 days of clopidogrel treatment, the mice were anesthetized (pentobarbital, 40 mg/kg and xylazine, 2.5 mg/kg, intramuscularly). Several minutes later, the mice were immobilized in a restraint device and had the tail cut 4 mm from the tip and immersed in 5 mL of distilled water at room temperature. The shed blood was evaluated as a function of the absorbance at 540 nm due to the hemoglobin content in the water solution. The concentration of hemoglobin was measured spectrophotometrically using a microplate spectrophotometer at 540 nm (Molecular Probes). The mice were then carefully observed for the next 6 h and checked the next morning for signs of delayed bleeding.

### Statistical analysis

Statistical analysis was performed with Prism software (GraphPad Software, Inc., San Diego, CA, USA). Results were analyzed by one-way ANOVA, followed by the Newman-Keuls test or by Student’s *t* test. Values are expressed as the mean ± standard error (SE). *P* values < 0.05 were considered significant.

## Results

### P2Y12R mediates ADP-induced eosinophil peroxidase (EPO) secretion in human eosinophils

**Fig 1:**
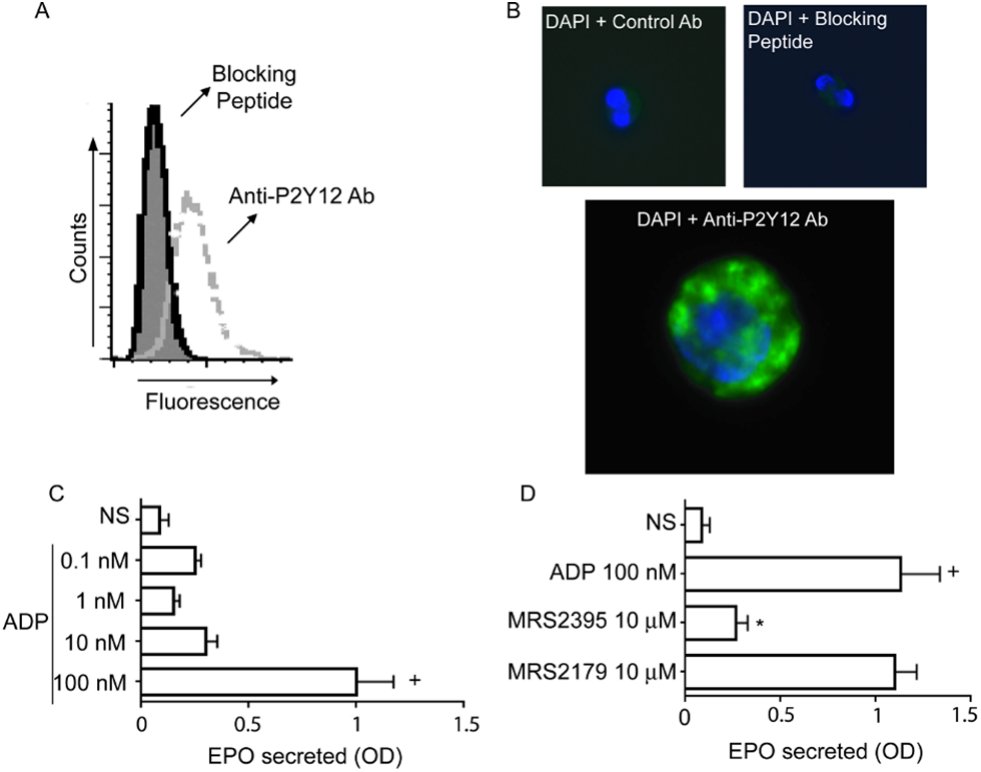
P2Y12R is expressed in human eosinophils and mediates ADP-induced eosinophil peroxidase (EPO) secretion. Analysis of the expression of P2Y12R in human eosinophils by (A) flow cytometry and (B) immunofluorescence. P2Y12R, green; nuclei, blue (after staining with DAPI). Shaded histogram represents staining with control antibody (Ab). Dashed and solid lines represent staining with polyclonal antibodies (pAbs) against P2Y12R and anti-P2Y12R pAbs neutralized by absorption with their immunogen peptide, respectively. Data are from one experiment, representative of 3 experiments. (C) ADP-induced EPO secretion after 1 h of stimulation is mediated by (D) P2Y12R. Graphs represent the mean ± SE (*n* = 3). Results were analyzed by one-way ANOVA, followed by the Newman-Keuls test (+ and *p<0.05 for EPO released compared with non-stimulated and ADP-stimulated eosinophils, respectively). NS = not stimulated, OD = optical density.

In a previous study, we characterized the expression of P2Y12R on human eosinophils [12]. In this study, we first conducted a series of experiments to confirm these findings. As shown in Fig 1A, human eosinophils exhibited immunoreactivity against the intracellular ligand-binding regions of P2Y12R. The specificity of the polyclonal antibody for P2Y12R was corroborated by complete neutralization of the immunostaining through preincubation of the anti-P2Y12R polyclonal antibody with its respective specific blocking peptide immunogen. Furthermore, we analyzed these P2Y12R-labeled eosinophils by fluorescence microscopy after DAPI (a nuclei marker) addition (Fig 1B). Human eosinophils showed immunoreactivity against P2Y12R, which suggests a cytoplasmic labeling. In fact, the expression of P2Y12R on isolated eosinophilic granules has been demonstrated previously [12]. To investigate the functional roles of the expression of P2Y12R on human eosinophils, we stimulated them with the nucleotide ADP (0.1–100 nM), a classical ligand of P2Y12R. The eosinophils responded to ADP (100 nM) (Fig 1C) by secreting granule-derived eosinophil peroxidade (EPO), indicating that the expression of P2Y12R on human eosinophils might have important functional roles in inducing eosinophil protein release. To investigate whether the ADP-induced EPO secretion was related to P2Y12R or P2Y1R activation, the isolated eosinophils were pretreated with P2Y12R (MRS2395) (10 μM) or P2Y1R (MRS2179) (10 μM) antagonists and then stimulated with ADP (100 nM). P2Y1R is also sensitive to ADP, and its expression in human eosinophils is already recognized [18]. As shown in Fig 1D, EPO secretion was significantly reduced in the presence of the P2Y12R blocker, but no significant difference was observed when the cells were pretreated with the P2Y1R antagonist.

### P2Y12R blockade reduces the area of the hepatic inflammatory infiltrate in a murine model of *S*. *mansoni* infection

**Fig 2:**
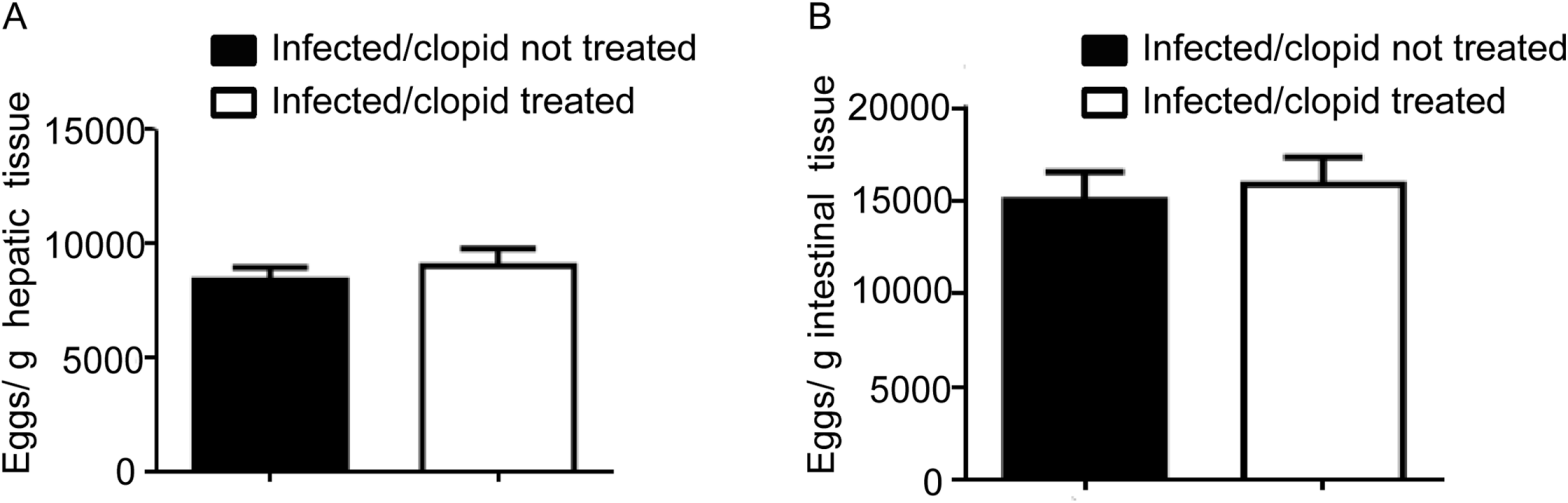
P2Y12R blockade does not alter the course of *S*. *mansoni* parasite oviposition. Mice were infected with 60 *S*. *mansoni* cercariae through a percutaneous route, and the livers and intestines were collected 55 days post-infection. Graphs show the number of eggs per gram tissue within (A) the liver and (B) intestines. Data represents the mean ± SE; 5–8 infected mice were used as individual tissue donors and represents animals from at least 2 independent infection studies.

**Fig 3:**
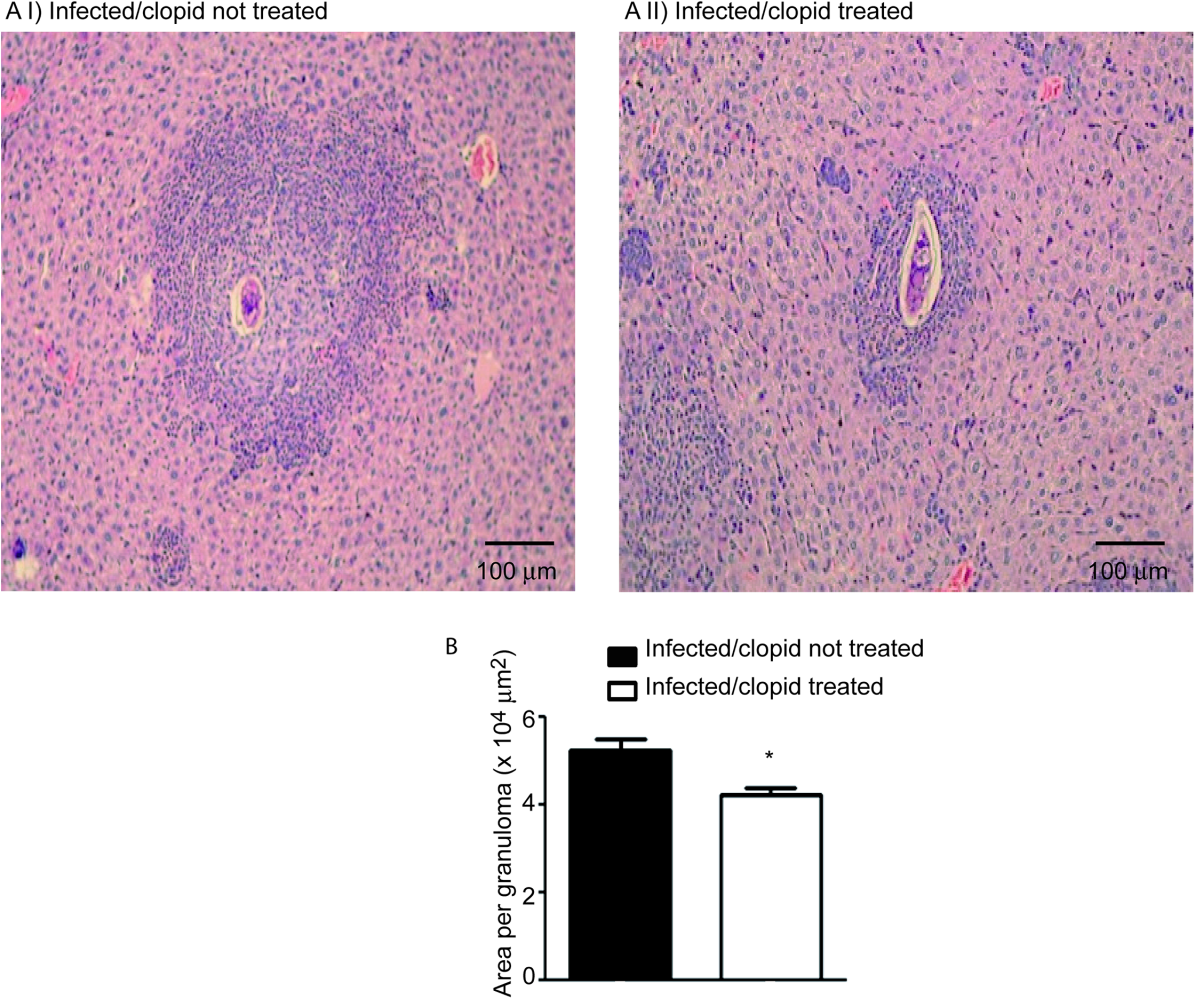
P2Y12R blockade reduces the area of the hepatic inflammatory infiltrate in a murine model of *S*. *mansoni* infection. Representative granuloma images from infected mice (Ai) untreated or (Aii) treated with clopidogrel. Slide sections were stained with hematoxylin-eosin and examined under a light microscope. (B) The individual granuloma area was estimated from 10–20 granulomas acquired for each individual mouse. Data represent the mean ± SE and were analyzed by Student’s *t* test. (*p<0.05). *n* = 5–8 mice/group and represents animals from at least 2 independent infection studies.

**Fig 4:**
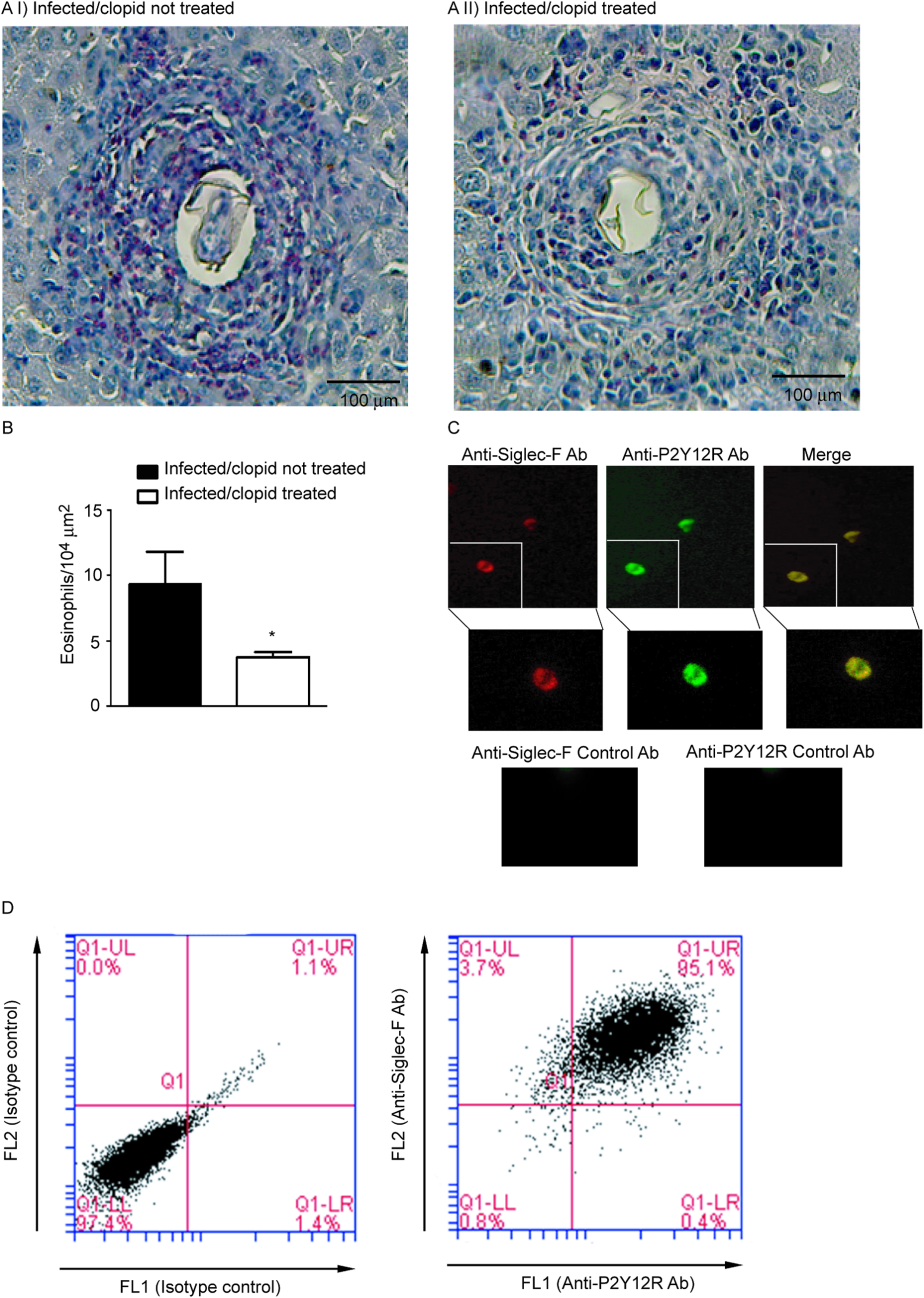
P2Y12R blockade impairs hepatic granulomatous eosinophilia. Representative granuloma images from infected mice (Ai) untreated or (Aii) treated with clopidogrel. Slide sections were stained with Syrius red (modified method) and examined under a light microscope. (B) The number of eosinophils per granuloma area were calculated from images of 10–20 granulomas acquired for each individual mouse. Data represent the mean ± SE and were analyzed by Student’s *t* test. (*p<0.05). *n* = 10 mice/group and represents animals from at least 2 independent infection studies. (C) Immunofluorescence showing Siglec-F and P2Y12R staining of cytospin smears of murine eosinophil-enriched suspensions. P2Y12R, green; Siglec-F, red; Ab = antibody. Data are from one experiment, representative of 3 experiments. (D) Flow cytometry dot plots showing isotype control (left panel) and Siglec-F versus P2Y12R staining (right panel) of mice bone marrow derived eosinophils. Data are from one experiment, representative of 3 experiments.

**Fig 5:**
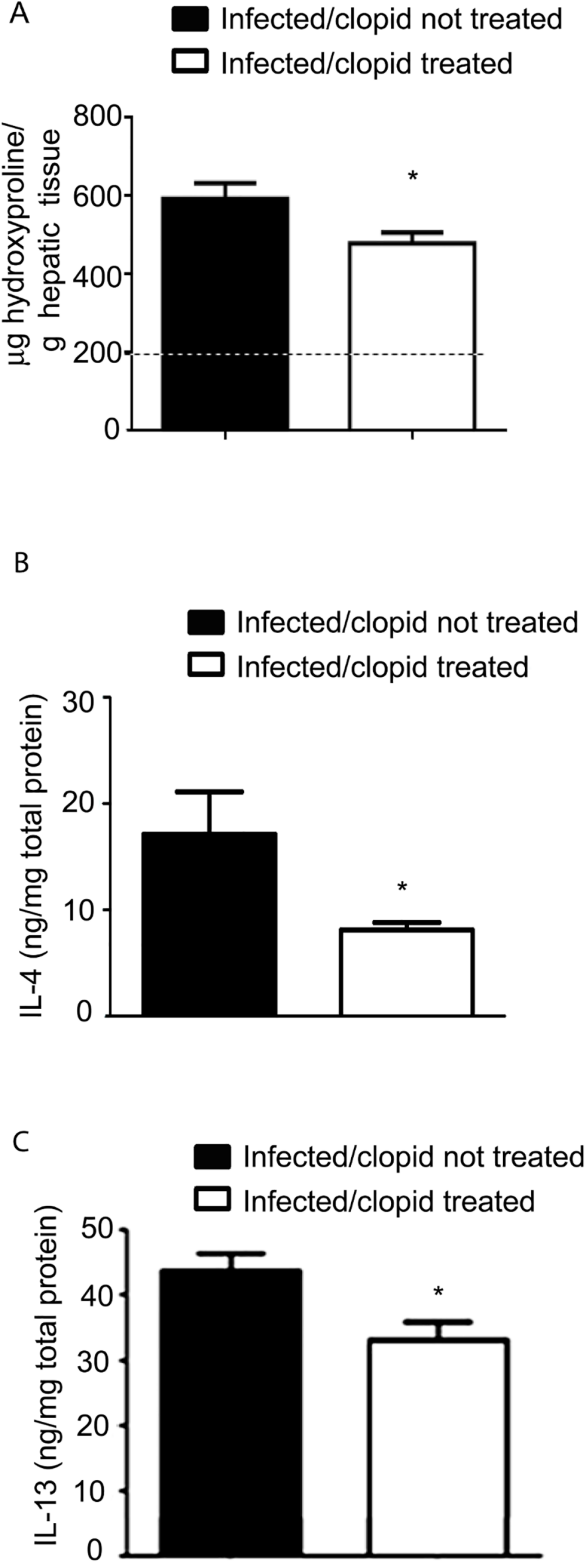
P2Y12R blockade reduces fibrosis and Th2 cytokine production in liver homogenates. (A) Liver collagen content, as assessed by hydroxyproline quantification, (B) IL-4 and (C) IL-13 were reduced in clopidogrel-treated mice compared to the untreated mice. Data represent the mean ± SE and were analyzed by Student’s *t* test. (*p<0.05). *n* = 5–8 mice/group and represents animals from at least 2 independent infection studies. Dashed line–hydroxyproline levels in uninfected mice.

The importance of the *in vivo* modulation of P2Y12R in the context of schistosomal inflammation was evaluated. We utilized a model of *S*. *mansoni* in mice, which causes a robust type 2 immune response. *S*. *mansoni* infection is known to promote a dramatic increase in liver fibrosis and in eosinophilopoesis, and in the number of eosinophils in the peripheral blood and granulomas in host tissue [4]. To investigate the role of P2Y12R in tissue granulomatous inflammatory reaction, we infected C57Bl/6 mice via the percutaneous route with 60 cercariae and analyzed their livers 55 days after infection. The animals were treated with a P2Y12R antagonist, clopidogrel (500 μg/mL) via the drinking water three days before and throughout the infection period (55 days). To assess the bioavailability of clopidogrel given in the drinking water, we performed a tail bleeding test based on the fact that clopidogrel is clinically used as an antiplatelet drug [19] (S1 Fig). The eggs present in the liver were counted 55 days postinfection. Similar numbers of eggs were found in both clopidogrel-treated and non-treated mice after liver (Fig 2A) and intestine (Fig 2B) analysis, thus indicating that the blockage of P2Y12R did not interfere with the control of parasite oviposition. The presence of *S*. *mansoni* eggs in the tissues triggered the formation of granulomas, characterized by the presence of macrophages, lymphocytes, and eosinophils [4]. Histopathological analysis of liver revealed that the granuloma sizes in the livers were macroscopically reduced in clopidogrel-treated mice (Fig 3AI and 3AII). A quantitative analysis was performed and revealed a significant difference between the treated and non-treated groups (Fig 3B). The number of eosinophils in the granulomas of the clopidogrel-treated group was also significantly reduced compared to the control group (Fig 4AI, 4AII and 4B). As found in human eosinophils, murine eosinophils from the liver of *S*. *mansoni*-infected mice also expressed the P2Y12R (Fig 4C), as assessed by immunofluorescence after analyzing Siglec-F and P2Y12R positive cells in cytospin smears. Using flow cytometry studies, we verified that more than 90% of murine bone marrow derived eosinophils express both Siglec-F and the P2Y12R (Fig 4D). Additionally, liver collagen content, as assessed by hydroxyproline quantification, was reduced in clopidogrel-treated compared with non-treated mice (Fig 5A). Similarly, the IL-4 and IL-13 cytokine levels, evaluated by ELISA in liver homogenates, were significantly reduced in the clopidogrel-treated group (Fig 5B and 5C), likely reflecting an attenuated inflammatory cell migration to the hepatic tissue.

### Th2 response to *S*. *mansoni* is not altered by P2Y12R blockade

**Fig 6:**
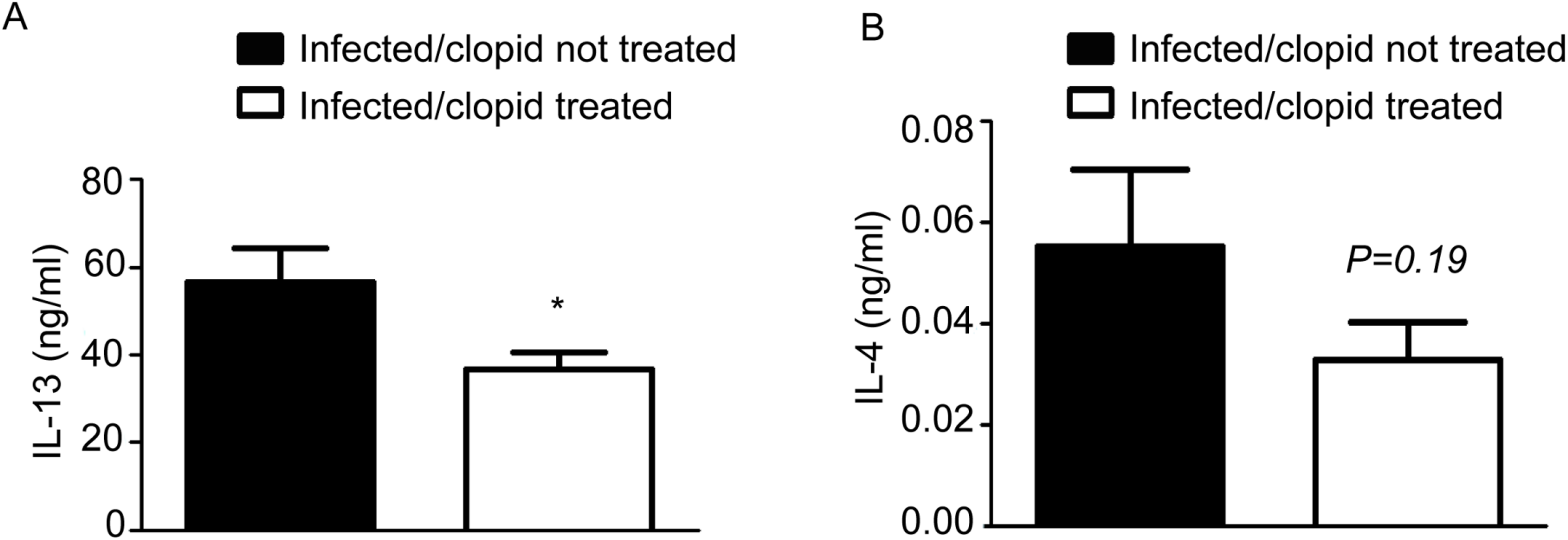
Th2 response to *S*. *manoni* infection is not altered by P2Y12R blockade. *S*. *mansoni-*infected, clopidogrel-treated mice displayed a slight difference in plasma concentrations of (A) IL-13, but no difference in (B) IL-4 levels was detected. Data represent the mean ± SE and were analyzed by Student’s *t* test. (*p<0.05). *n* = 5–8 mice/group and represents animals from at least 2 independent infection studies.

Deficiency of Th2 polarization can inhibit eosinophilopoiesis and reduce the size of the granuloma on *S*. *mansoni* infection, which occurs in IL-5 or in concomitant IL-13/IL-4 deficiencies [25, 26]. Therefore, we verified whether P2Y2R blockade could alter Th2 polarization and thus impair eosinophil-dependent inflammation. *S*. *mansoni* infected, clopidogrel-treated mice displayed a slight difference in the plasma concentrations of IL-13 (Fig 6A) but no difference in the IL-4 levels (Fig 6B). Furthermore, when spleen cells were cultured with schistosomal egg antigen (SEA) *in vitro* for 24 h and 48 h, the secreted levels of IL-13 were similar or slightly greater in clopidogrel-treated mice compared with non-treated controls (data not shown).

### P2Y12R blockade promotes an increase in blood eosinophilia but decreases the eosinophil count in the bone marrow

**Fig 7:**
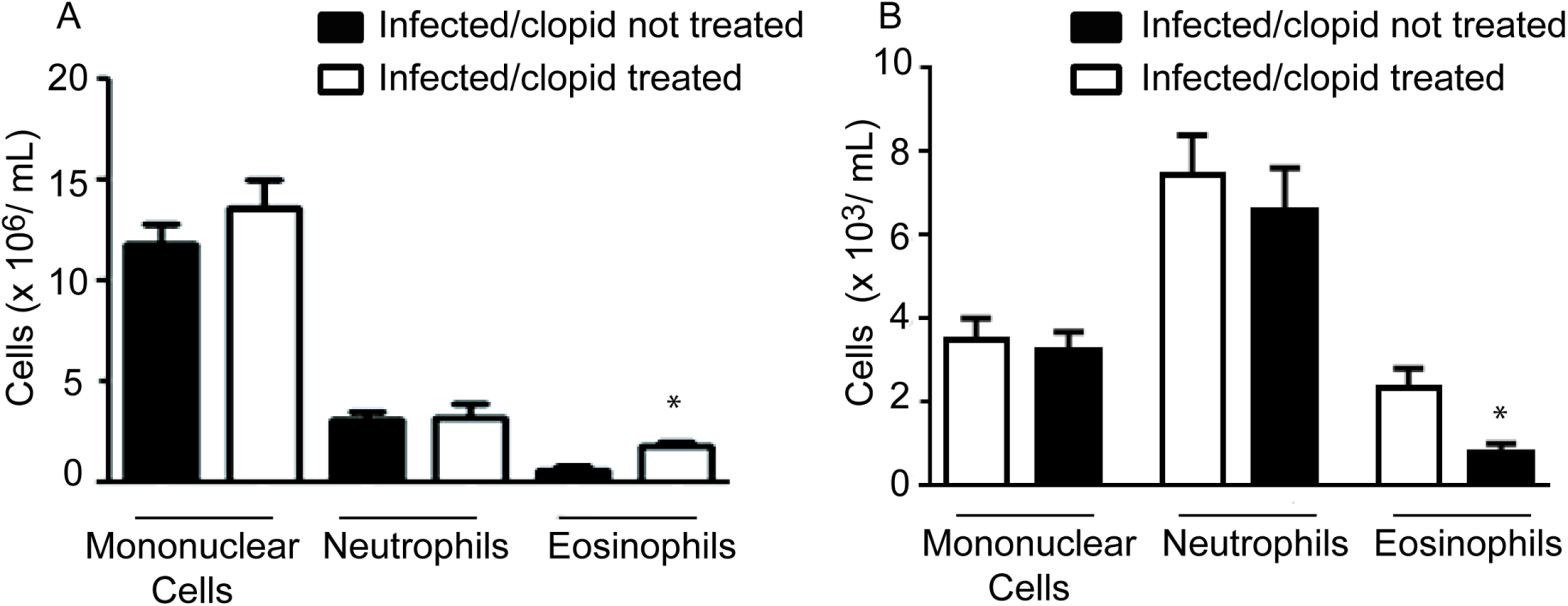
P2Y12R blockade promotes an increase in blood eosinophilia but decreases the eosinophil count in the bone marrow. (A) The number of eosinophils was estimated in the blood of infected, clopidogrel-untreated and clopidogrel-treated mice (Panoptic kit-stained smears). (B) The number of eosinophils in the individual bone marrow was evaluated in clopidogrel-untreated and -treated mice (Panoptic kit-stained cytospins). Data represent the mean ± SE and were analyzed by Student’s *t* test. (*p<0.05). *n* = 5–8 mice/group and represent animals from at least 2 independent infection studies.

*S*. *mansoni* infection causes robust eosinophilopoiesis and blood eosinophilia [8]. To investigate whether blocking P2Y12R could interfere with these parameters, we performed blood smears and a bone marrow count of clopidogrel-treated and untreated animals. Treatment with a P2Y12R blocker increased the number of eosinophils in the blood (Fig 7A), whereas it reduced eosinophil numbers in the bone marrow (Fig 7B). No changes were observed for mononuclear cells or neutrophils. These results might suggest that blocking P2Y12R may influence the recruitment of eosinophils in the bone marrow into the blood or impair the migration of these cells into the infected tissues.

### ADP does not act as a chemotactic for human eosinophils and neither induces or prevents their survival

**Fig 8:**
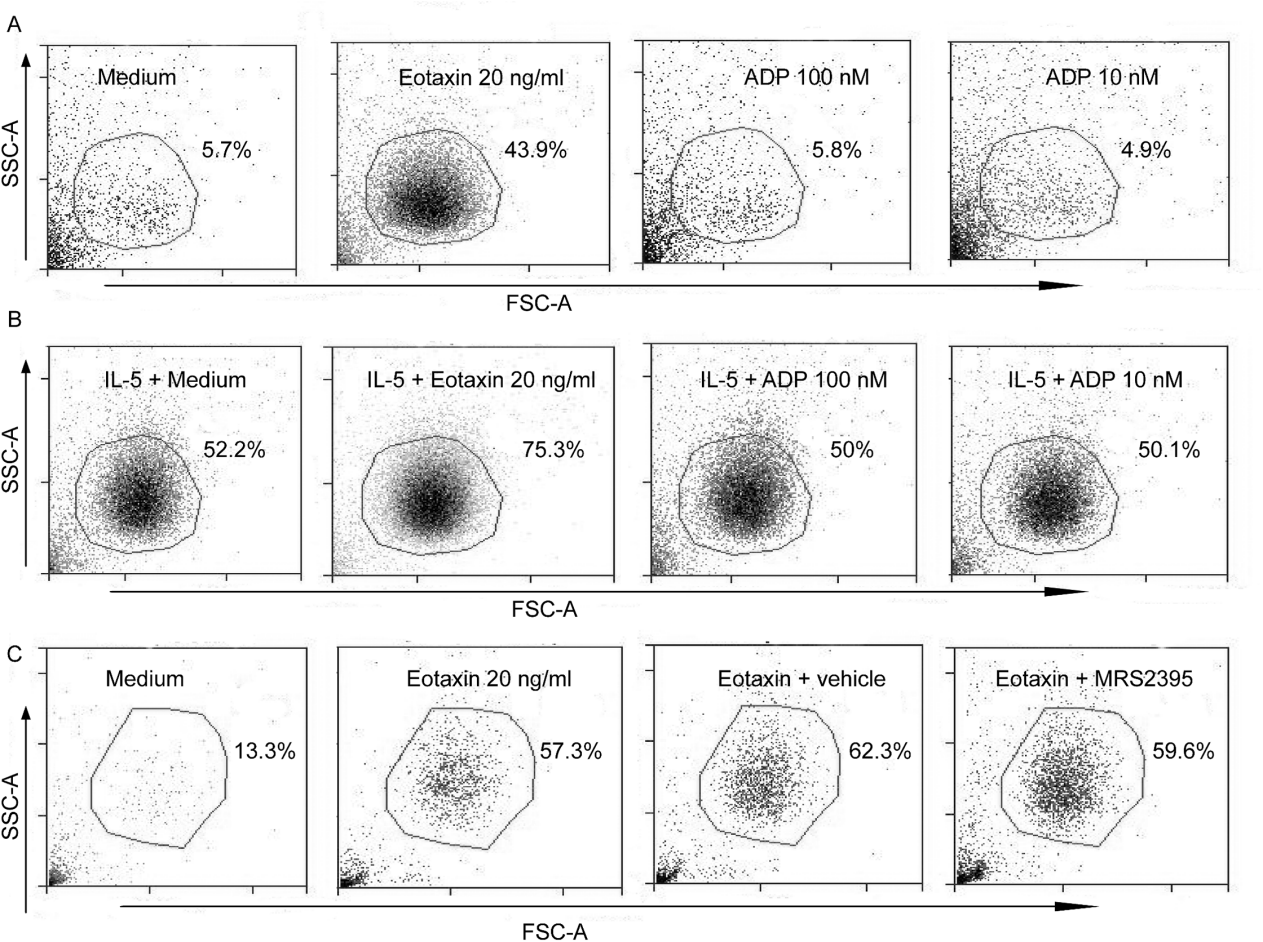
ADP is not chemotactic for human eosinophils. Human eosinophil-enriched suspensions containing 3x10^5^ cells/100 μL were left to migrate in transwell plates against eotaxin (20 ng/mL) (positive control) or ADP (100 and 10 nM) in (A) the absence or (B) presence of IL-5 (30 ng/mL) for 3 h at 37°C. (C) MRS2395 (10 μM), a P2Y12R antagonist, failed to inhibit the eotaxin-induced eosinophil recruitment. Data are representative dot plots (SSC-A/FSC-A) of 3 independent experiments (n = 3). Vehicle = MRS2395 diluent (DMSO).

**Fig 9:**
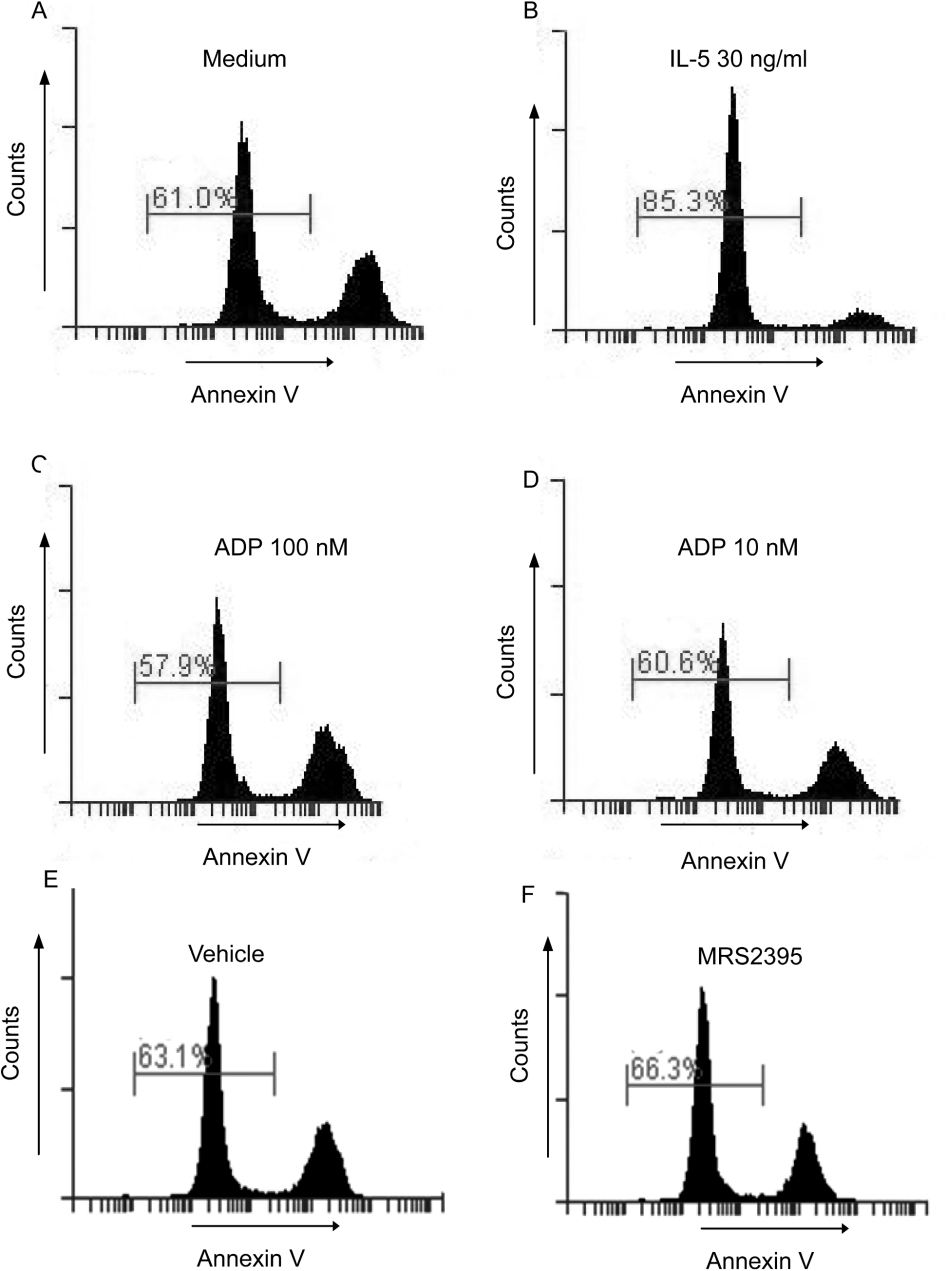
ADP did not induce/prevent eosinophil apoptosis. Human eosinophils were cultured for 24 h in the presence of IL-5 (30 ng/mL) or ADP (100 and 10 nM) or MRS2395 (10 μM), followed by labeling with annexin V and flow cytometry analysis. Data are representative histograms of 3 independent experiments (n = 3). Vehicle = MRS2395 diluent (DMSO).

Because liver granulomas from *S*. *mansoni-*infected, clopidogrel-treated mice had fewer eosinophils compared with those from untreated mice, we investigated whether ADP could also be a chemoattractant for eosinophils or prevent eosinophil apoptosis. It has been reported that nucleotides such as ATP and ADP at high, non-physiological concentrations have a chemoattractant effect on neutrophils, eosinophils and macrophages [15, 18, 27, 28]. Human isolated eosinophils were submitted to a transwell assay against eotaxin (20 ng/ml), a known eosinophil chemoattractant (used here as a positive control), and ADP (100 and 10 nM). Eotaxin attracted large numbers of eosinophils (43.9%) compared to non-stimulated conditions (medium) (5.7%)(Fig 8A). However, ADP at concentrations capable of inducing EPO secretion (100 and 10 nM) failed to promote eosinophil chemotaxis (5.8% and 4.9%, respectively) compared to medium (5.7%)(Fig 8A). To evaluate whether P2Y12R might be required for optimal IL-5-induced chemokinetic responses, eosinophils were primed with IL-5 for 1 h. It is known that IL-5 priming induces random, chemokinetic migration of eosinophils [29] and enhances eosinophil chemotactic responses to subsequent stimulation (i.e., with IL-8 or leukotriene B_4_ [LTB_4_]) *in vitro* [29]. As shown in Fig 8B, ADP (100 and 10 nM) did not interfere with eosinophil migration after IL-5 priming (50% and 50.1%, respectively) compared to the condition without ADP (IL-5 + medium) (52.2%). Eotaxin (used here as a positive control) showed enhanced eosinophil chemoattractant properties (75.3%) compared to just IL-5 priming (IL-5 + medium) (52.2%) (Fig 8B) or eotaxin alone (43.9%). Next, we investigated whether P2Y12R might be involved in chemotaxis induced by eotaxin, as previously mentioned, a known physiologic eosinophil chemoattractant. As shown in Fig 8C, the compound MRS2395 10 μM (a P2Y12R blocker) failed to impair eotaxin-induced eosinophil recruitment (59.6%) compared to the control (eotaxin + vehicle) (62.3%). To assess a possible anti-apoptotic effect for ADP in human eosinophils, the cells were cultured 24 h with ADP (100 and 10 nM), labeled with annexin V and immediately evaluated by flow cytometry. No difference in eosinophil apoptosis was observed when the cells were cultured with 100 nM (Fig 9C) or 10 nM ADP (Fig 9D) compared with the control conditions (Fig 9A). Eosinophils cultured for 24 h with IL-5 were used as a positive control (Fig 9B). This result suggests that P2Y12R does not interfere with the apoptosis of human eosinophils. The compound MRS2395 (10 μM), a P2Y12R antagonist, does not have apoptotic effects itself (Fig 9F) compared to its diluent (DMSO) (Fig 9E).

## Discussion

Identifying new target molecules that account for eosinophil activation may be highly important for our understanding of the pathophysiology of host immune responses to parasites, allergic inflammation and related eosinophilic diseases. We have recently reported the expression of P2Y12R on human eosinophils and isolated human eosinophil granules [12]. In the present study, we demonstrated that isolated human eosinophils secrete EPO in response to ADP in a mechanism dependent on P2Y12R. *In vivo*, we found that P2Y12R blockade reduces the hepatic granulomatous inflammatory response in *S*. *mansoni* infection without affecting the Th2 response. P2Y12R blockade modulated hepatic tissue inflammation but did not impair *S*. *mansoni* egg oviposition, indicating that the blockage of P2Y12R does not affect the infection regarding parasite load. In this regard, clopidogrel-treated mice had reduced granuloma areas with less eosinophils during *S*. *mansoni* infection. However, the plasma levels of IL-13 and IL-4, as well as the levels of IL-13 produced by splenocytes in response to SEA, were not importantly affected by P2Y12R blockage, indicating that P2Y12R is not essential to Th2 polarization. As found for humans, murine eosinophils also express the P2Y12R. Because liver granulomas from *S*. *mansoni-*infected, clopidogrel-treated mice had fewer eosinophils compared with those from untreated mice, we tested whether ADP could also be a chemoattractant to eosinophils or prevent eosinophil apoptosis. We found that this is unlikely based on our results *in vitro*, in which ADP did not interfere with eosinophil chemotaxis or survival. Our results demonstrate a previously unrecognized role for P2Y12R in as a modulator of eosinophil activation and in schistosomal inflammation.

Using flow cytometry and immunofluorescence techniques, our results confirmed previous findings that demonstrate that peripheral human eosinophils express P2Y12R. Notably, immunofluorescence microscopy images indicate an intracellular clustered labeling for P2Y12R. In fact, our group previously described P2Y12R expression in intact, non-permeabilized eosinophils and on the membranes of intracellular eosinophil granules [12]. Functionally, our results show that P2Y12R is involved in EPO secretion by eosinophils. Data from the literature indicate that other cationic proteins, such as ECP, and cytokines such as IL-8 are secreted in response to ATP, UTP and UDP, but not ADP [30]. Notably, in this study, the cells were stimulated with nucleotides at micromolar concentrations, contrasting with the nanomolar range used in our experiments. Studies suggest that eosinophils can cause parasite harm in *S*. *mansoni* and other worm infections *in vitro* [5] and *in vivo* [6]. Nevertheless, mice that are selectively deficient in eosinophils [9] or IL-5 [25] control parasite burden as efficiently as wild-type mice. In agreement with these data, we showed here that *S*. *mansoni* infection in clopidogrel-treated mice is efficiently controlled concerning the parasite load, despite the reduced numbers of eosinophils. In our findings we noticed a prominent reduction in the area size of granulomas possible due to the reduction of infiltrating inflammatory cells. Despite we identified less eosinophils in the granulomas and found that mouse eosinophils also express the P2Y12R, whether they or other cells are responsible to the observations related to granuloma size is not possible to define. In addition, if mouse eosinophils are capable to secrete cationic proteins *in vivo* via the P2Y12R activation and contribute to the inflammatory host response is still not known. The granuloma formation around eggs also depends on Th2 responses, as demonstrated in IL-4/IL-13- [26], IL-10/IL-4- [31], and IL-4αR-deficient mice [32], in which the development of Th2-polarized responses to eggs leads to abnormal granulomas and liver injury. The reduction in the area size of the liver granulomas in *S*. *mansoni*-infected, clopidogrel-treated mice does not seem to be the consequence of a weakened Th2 response because infected clopidogrel-treated mice do not have strongly reduced plasma concentrations of IL-4 and IL-13 and do not have lower levels of IL-13 production by spleen cells in response to SEA. These results indicate that the control exerted by the P2Y12R blockade on the area size of egg granulomas is likely not related to a disorder of the Th2 response.

The production of IL-13 [26] and tissue eosinophilia have been thought to participate in the fibrosis and resultant portal hypertension that causes morbidity during the chronic phase of the infection. Our results of collagen deposition in *S*. *mansoni*-infected mice showed that P2Y12R blockade is relevant for liver fibrosis induced by Th2 inflammation. One hypothesis is that the reduced inflammatory cells infiltration might be contributing to the impaired collagen deposition in clopidogrel-treated mice compared with the non-treated group. The role of eosinophils in promoting fibrosis and collagen deposition remains controversial. Although a previous study demonstrated an essential role for eosinophils on the collagen deposition and tissue remodeling induced by OVA immunization and challenge using eosinophil-ablated mice [33], a collagen deposition similar to wild-type controls was found in the livers of these eosinophil deficient mice when they were infected with *S*. *mansoni* [9]. Because several studies have confirmed that the production of IL-13, in contrast, is a principal factor inducing fibrosis [25, 26], we believe that the impaired hepatic and plasma levels of IL-13 that we found in clopidogrel-treated compared to clopidogrel-untreated mice can account for their diminished collagen deposition.

The reduced granuloma sizes at *S*. *mansoni*-infected sites might be associated with impaired leukocyte transmigration from the vascular compartment. *S*. *mansoni* infection causes robust eosinophilopoiesis and blood eosinophilia [8]. In this regard, the analysis of blood and bone marrow eosinophilia in clopidogrel-treated mice revealed that P2Y12R blockade promoted an increase in blood eosinophilia but decreased the eosinophil count in the bone marrow. No changes were observed for mononuclear cells or neutrophils. These results might suggest that blocking P2Y12R may influence the recruitment of eosinophils from the bone marrow to the blood or impair the migration of these cells to the infected tissues. There is no evidence in the literature that correlates P2Y12R and modulation of adhesion molecule expression or eosinopoiesis. However, the expression and the role of P2Y12R in platelets as therapeutic targets for antiplatelet agents is widely known [34]. Beyond their primary role in hemostasis, platelets are causally involved in the onset of inflammatory reactions, cell proliferation and the immune response [35]. Platelet activation and platelet binding to the endothelium result in the release of chemokines and the increased expression of adhesion molecules, which promote the recruitment of leukocytes that will eventually migrate across the endothelium into the tissue [35]. Thus, a reduction in eosinophil transmigration might be a consequence of the impairment of platelet aggregation in clopidogrel-treated mice. Furthermore, through their ability to interact with other cells, platelets are involved in many physiological and pathological processes in addition to vascular inflammation, including eosinophilic disorders such as allergic asthma [36, 37]. Platelets are necessary for lung leukocyte recruitment in a murine model of allergic inflammation [38]. In addition, platelets and P2Y12R has been reported to be required for the inflammatory activities of the stable abundant mediator LTE4 in asthma and has been suggested to be a potential therapeutic target for this disease because clopidogrel showed efficacy in the treatment of experimental allergen-induced pulmonary inflammation [20]. Although not fully elucidated, the mechanism suggested for the role of P2Y12R in this process was its association with an as yet not identified coreceptor of the cysteinyl leukotriene family. Whether platelets or their activation via the P2Y12R are important for the inflammatory host responses to *S*. *mansoni* is still unknown. P2Y12R may also be expressed in other cells of the immune system involved in the inflammatory host responses to *S*. *mansoni*. High amounts of P2Y12R mRNA have been found previously in both lymphocytes and CD34+ progenitor cells [39]. In addition, a varied range of purinergic receptors is expressed in dendritic cells and plays roles in their maturation and the coordination of the immune response [40]. In these cells, ADP stimulates calcium mobilization and ERK phosphorylation in a pertussis toxin-dependent manner as well as inhibits IL-12 production [40]. However, it is important to notice that the use of clopidogrel in millions of patients with ischemic diseases over the years has not yet been stated to cause the development of selective immune disorders related to the drug. Similarly, patients deficient in the P2Y12R do not suffer from explicit disorders of their immune system [36].

Because we demonstrated that P2Y12R is important for modulating the schistosomal inflammatory response and tissue eosinophilia, we wondered whether ADP would be capable of attracting human eosinophils in transwell assays as well as acting as a survival factor for these cells. ADP selectively activates the receptors for P2Y1, P2Y12 and P2Y13. ADP is known to activate P2Y1R, P2Y12R and P2Y13R, with EC_50_ values of 8 μM, 0.07 μM and 0.06 μM, respectively [41]. Human eosinophils are recognized to express P2Y1R and P2Y12R [18]. Thus far, there is no evidence of the expression of P2Y13R in human eosinophils. Thus, the use of ADP at nanomolar concentrations in our experiments might be expected to selectively act on P2Y12R. Nucleotides belong to the group of constitutive DAMPs that are released as a consequence of cellular damage during the course of infection or in sterile inflammation [42]. Because of its small size and high mobility, ATP can be fast released together with other cellular components after cell injury, mechanical stress or cell death [42]. Augmented ATP concentration in the extracellular milieu is thus closely associated with tissue stress or damage [42]. However, nonlytic nucleotide release may happen in different cell types under a diversity of conditions. ATP is found at relatively high levels in the cytoplasm of cells, where its concentration ranges from 1 to 10 mM. In the extracellular space, its physiologic concentration is significantly lower, ranging between 1 and 10 nM [43]. Once released extracellularly, ATP is rapid converted to ADP, AMP and adenosine by cell membrane-expressed ectonucleotidases [43]. In our studies, ADP at concentrations capable of inducing eosinophil EPO secretion did not promote or prevent eosinophil apoptosis. There is no evidence in the literature concerning the effect of nucleotides or purinergic receptors on eosinophil survival. Furthermore, ADP at concentrations capable of inducing eosinophil EPO secretion was not chemotactic for human eosinophils. Different studies suggest that nucleotides such as ATP and ADP are chemotactic for eosinophils [44–46] and other cell types [27, 47]. Burgers and colleagues suggest that ATP secreted by platelets activated by thrombin activates and is chemotactic for human eosinophils [44]. These studies were followed by others in which ADP, ATP and other nucleotides were shown to be capable of inducing eosinophil chemotaxis, ROS production and CD11b expression [17, 18, 45, 48]. However, in all of these studies, the concentrations of ADP used were at micromolar concentrations or higher, which might be considered a supraphysiological range. Our concern is whether the utilization of abnormal nucleotide concentrations might alter the physiological conditions, resulting in artificial responses and in the loss of ADP specificity in purinergic receptors. In fact, different studies describe the chemotactic effects of ATP, ADP and other nucleotides in different immune cells, including neutrophils, mast cells, lymphocytes, dendritic cells and macrophages [17]. However, in all of these studies, the concentrations of nucleotides utilized were at a higher range than we used in our studies.

IL-5 priming induces the random, chemokinetic migration of eosinophils [29] and enhances eosinophil chemotactic responses to subsequent stimulation (i.e., with IL-8 or leukotriene B4) *in vitro*. However, even after IL-5 priming, ADP (100 and 10 nM) was not capable of promoting human eosinophil chemotaxis. We also verified possible autocrine/paracrine roles of P2Y12R in the chemotaxis induced by known eosinophil physiological activators such as eotaxin. A study published by Kronlage and colleagues revealed that C5a-induced macrophage chemotaxis is dependent on the paracrine/autocrine activation of the P2Y12 and P2Y2 receptors [27]. Additionally, Isford and colleagues described that the activation of these receptors is important for macrophage chemokinesis, but not chemotaxis, contrasting the data that show that nucleotides work as a “find me” signal for macrophages, directing them toward inflamed sites [49]. In our experiments, P2Y12R antagonists failed to block eotaxin-induced eosinophil migration, suggesting that this receptor is not likely involved in the chemotactic response induced by this chemokine.

In conclusion, our studies demonstrate that P2Y12R modulation plays a role in eosinophil biology and in the host response to schistosomal infection. The reduced granulomatous area in clopidogrel-treated mice during *S*. *mansoni* infection occurred irrespective of robust alterations in the antigen-driven immune response, thus indicating that the response mediated by this receptor is downstream of these events. Our data also implicate P2Y12R as a mediator for ADP-induced EPO secretion in human eosinophils, but not for eosinophil chemotaxis or survival. Together, these results demonstrate a formerly unrecognized role for P2Y12R in eosinophil activation and schistosomal inflammation.

## Supporting Information

S1 Fig Bioavailability of clopidogrel given in the drinking water, as assessed by the tail bleeding test. The concentration of hemoglobin was measured spectrophotometrically using a microplate spectrophotometer at 540 nm. Data represent the mean ± SE and were analyzed by Student’s *t* test. (*, p<0.05). n = 5–8 mice/group and represents animals from at least 2 independent infection studies. OD = optical density.
(PDF)
