# Supplementary figures and images for: Purinergic P2Y12 Receptor Activation in Eosinophils and the Schistosomal Host Response
Source: PLoS One. 2015 Oct 8;10(10):e0139805. doi: 10.1371/journal.pone.0139805 (PMC4598158; doi:10.1371/journal.pone.0139805)

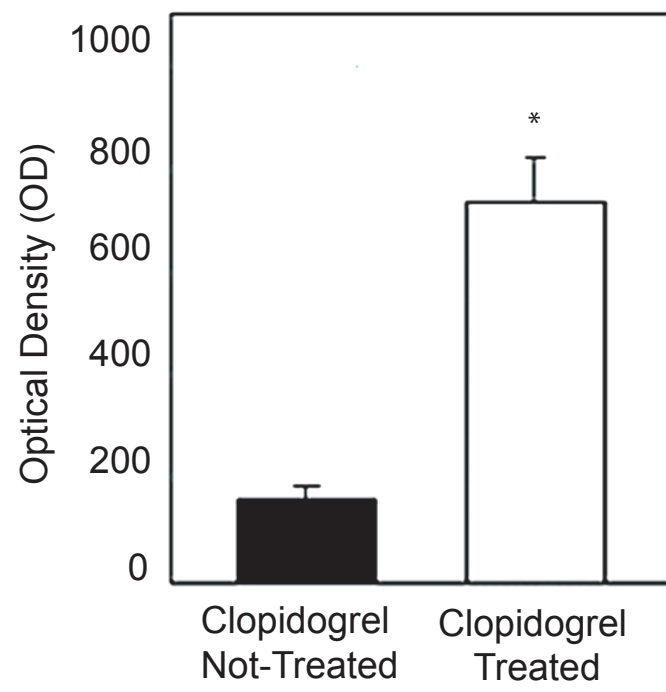

Supplement: S1 Fig — The concentration of hemoglobin was measured spectrophotometrically using a microplate spectrophotometer at 540 nm. Data represent the mean ± SE and were analyzed by Student’s t test. (*, p<0.05). n = 5–8 mice/group and represents animals from at least 2 independent infection studies. OD = optical density. (PDF) [file pone.0139805.s001.pdf]
